# Secondary analysis of the EMPACT-MI trial reveals cardiovascular–kidney efficacy and safety of empagliflozin after acute myocardial infarction

**DOI:** 10.1038/s44161-025-00657-7

**Published:** 2025-06-13

**Authors:** Rahul Aggarwal, Deepak L. Bhatt, Adrian F. Hernandez, Stefan D. Anker, Josephine Harrington, W. Schuyler Jones, Michaela Mattheus, Mark C. Petrie, Dominik Steubl, Mikhail Sumin, Vikram Thanam, Jacob A. Udell, Javed Butler

**Affiliations:** 1 Brigham and Women’s Hospital Heart and Vascular Center, Harvard Medical School, Boston, MA USA; 2 Mount Sinai Fuster Heart Hospital, Icahn School of Medicine at Mount Sinai, New York, NY USA; 3 Duke University Department of Medicine, Division of Cardiology, and Duke Clinical Research Institute, Durham, NC USA; 4 Department of Cardiology (CVK) of German Heart Center Charité, Berlin Institute of Health Center for Regenerative Therapies (BCRT), German Centre for Cardiovascular Research (DZHK) partner site Berlin, Charité Universitätsmedizin, Berlin, Germany; 5 Boehringer Ingelheim Pharma GmbH & Co. KG, Ingelheim, Germany; 6 School of Cardiovascular and Medical Sciences, British Heart Foundation Glasgow Cardiovascular Research Centre, University of Glasgow, Glasgow, UK; 7 Department of Nephrology, Hospital rechts der Isar, Technical University Munich, Munich, Germany; 8 Boehringer Ingelheim International GmbH, Ingelheim, Germany; 9 Women’s College Hospital and Peter Munk Cardiac Centre, Toronto General Hospital, University of Toronto, Toronto, Ontario Canada; 10 Baylor Scott and White Research Institute, Dallas, TX USA; 11 Department of Medicine, University of Mississippi, Jackson, MS USA

**Keywords:** Cardiology, Medical research

## Abstract

Data on the cardiovascular–kidney effects and safety of empagliflozin among patients with acute myocardial infarction are limited. EMPACT-MI (Study to Evaluate the Effect of Empagliflozin on Hospitalization for Heart Failure and Mortality in Patients with Acute Myocardial Infarction) was a double-blind, multicenter clinical trial that randomized 6,522 patients with acute myocardial infarction and risk for heart failure to empagliflozin or placebo. Here we show in this secondary analysis that the mean estimated glomerular filtration rate at baseline was 76.1 ml min^−1^ 1.73 m^−^^2^ (s.d. = 19.9 ml min^−1^ 1.73 m^−^^2^), with longitudinal kidney function data available for 1,152 (17.7%) treated patients from select countries. By 24 months, compared with baseline, the estimated glomerular filtration rate was similar in the empagliflozin group but declined in the placebo group (*P* = 0.01). Empagliflozin reduced the total adverse events of heart failure or all-cause mortality irrespective of kidney function (*P*_interaction_ = 0.30). Thirty-day adverse event rates were similar by treatment group and consistent across baseline kidney function. Empagliflozin had kidney-protective effects, reduced heart failure outcomes and was safe to initiate soon after acute myocardial infarction across baseline kidney function.

## Abstract (web summary)

Aggarwal et al. report a secondary analysis of the EMPACT-MI trial, which evaluates the effects of empagliflozin according to baseline kidney function in patients after myocardial infarction, showing that empagliflozin offers kidney protection, reduces heart failure events and was safe to initiate in patients with acute myocardial infarction, irrespective of baseline kidney function.

## Main

In EMPACT-MI (Study to Evaluate the Effect of Empagliflozin on Hospitalization for Heart Failure and Mortality in Patients with Acute Myocardial Infarction), the sodium-glucose cotransporter 2 (SGLT2) inhibitor empagliflozin reduced heart failure (HF) events among patients with acute myocardial infarction (MI) and an increased risk for HF, although without improvement in all-cause mortality^1–4^. Patients with acute MI have a high burden of kidney dysfunction, with about 40% having chronic kidney disease (CKD)^5,6^, and high rates of acute decompensated HF^7,8^.

Data regarding the cardiovascular–kidney efficacy and safety of SGLT2 inhibitors among patients with acute MI with variable kidney function are limited. DAPA-MI (Dapagliflozin Effects on Cardiometabolic Outcomes in Patients With an Acute Heart Attack) was the only other clinical trial to evaluate SGLT2 inhibitors among patients with acute MI, but it had limited enrollment of patients with kidney dysfunction, few HF events and did not assess kidney outcomes^9^. Studies of patients with HF have shown the benefit and safety of initiating SGLT2 inhibitors early, among many patients at index hospital admission, but have not assessed the safety in patients with acute MI^10–16^. It is especially important to understand this safety profile among patients with acute MI because these individuals have high risk for acute kidney injuries (AKIs), substantial risk for volume depletion or hypotension and regular exposure to kidney stressors, such as contrast for imaging or cardiac catheterization or kidney-function-altering medications^6,17,18^.

To address this gap in evidence, we conducted a secondary analysis of EMPACT-MI and evaluated three questions. First, does initiation of empagliflozin early after acute MI affect kidney function? Second, does empagliflozin improve HF endpoints among patients with acute MI irrespective of baseline kidney function? Third, is empagliflozin initiation safe during or early after acute MI hospitalization, including among patients with varying kidney function and commonly co-prescribed therapies?

## Results

A total of 6,610 patients were screened from December 2020 to March 2023, with 6,522 patients randomized, including 3,260 (50.0%) to empagliflozin and 3,262 (50.0%) to placebo. Data on screen failure, early discontinuation and follow-up have been described previously^1^. The median time from index acute MI to randomization was 5 days (interquartile range (IQR) = 3–8 days) and the median duration of follow-up was 17.9 months. Median time from hospital admission for index MI to discharge was 5 days (3–8 days). Mean age was 63.6 years (s.d. = 10.9 years), with 1,625 (24.9%) females.

Among these patients, the mean estimated glomerular filtration rate (eGFR) was 76.1 ml min^−1^ 1.73 m^−^^2^ (s.d. = 19.9 ml min^−1^ 1.73 m^−^^2^). A total of 1,803 (27.6%) had an eGFR ≥90 ml min^−1^ 1.73 m^−^^2^, 3,261 (50.0%) ≥60 to <90 ml min^−1^ 1.73 m^−^^2^, 1,399 (21.5%) ≥30 to <60 ml min^−1^ 1.73 m^−^^2^ and 59 (0.9%) <30 ml min^−1^ 1.73 m^−^^2^. A total of 4,845 (74.3%) patients presented with ST-elevation MI (STEMI) and 1,675 (25.7%) with non-ST-elevation MI (NSTEMI), with 5,822 (89.3%) patients undergoing revascularization. A total of 1,886 (28.9%) patients received additional contrast administration in addition to coronary angiography or revascularization procedure. Clinical characteristics according to baseline eGFR category are shown in Table 1 and Supplementary Table 1.

**Table 1:** Baseline characteristics according to kidney function

Characteristic	Overall (*n* = 6,522)	eGFR ≥ 90 ml min^−1^ 1.73 m^−^^2^ (*n* = 1,803)	eGFR ≥60 to <90 ml min^−1^ 1.73 m^−^^2^ (*n* = 3,261)	eGFR ≥30 to <60 ml min^−1^ 1.73 m^−^^2^ (*n* = 1,399)	eGFR < 30 ml min^−1^ 1.73 m^−^^2^ (*n* = 59)
Age (years), mean (s.d.)	63.6 (10.9)	56.4 (9.6)	64.8 (9.9)	69.9 (9.5)	73.2 (9.4)
Female sex	1,625 (24.9)	312 (17.3)	775 (23.8)	508 (36.3)	30 (50.8)
Index MI type^a^					
STEMI	4,845 (74.3)	1,403 (77.8)	2,439 (74.8)	969 (69.3)	34 (57.6)
NSTEMI	1,675 (25.7)	400 (22.2)	821 (25.2)	429 (30.7)	25 (42.4)
Revascularization for index MI	5,822 (89.3)	1,651 (91.6)	2,955 (90.6)	1,166 (83.3)	50 (84.7)
Thrombolytic therapy for index MI	700 (10.7)	215 (11.9)	357 (10.9)	127 (9.1)	1 (1.7)
Contrast administration in addition to coronary angiography or revascularization procedure	1,886 (28.9)	505 (28.0)	954 (29.3)	412 (29.4)	15 (25.4)
Lowest LVEF^a^					
<25%	252 (3.9)	60 (3.3)	114 (3.5)	74 (5.3)	4 (6.8)
25–34%	1,420 (21.8)	446 (24.7)	688 (21.1)	269 (19.2)	17 (28.8)
35–44%	3,440 (52.7)	971 (53.9)	1,772 (54.3)	677 (48.4)	20 (33.9)
45–54%	906 (13.9)	203 (11.3)	442 (13.6)	248 (17.7)	13 (22.0)
≥55%	452 (6.9)	108 (6.0)	218 (6.7)	122 (8.7)	4 (6.8)
Signs or symptoms of congestion that resulted in treatment	3,715 (57.0)	888 (49.3)	1,798 (55.1)	983 (70.3)	46 (78.0)
Cardiovascular disease history and risk factors					
Previous MI	847 (13.0)	197 (10.9)	420 (12.9)	220 (15.7)	10 (16.9)
Atrial fibrillation	689 (10.6)	97 (5.4)	342 (10.5)	242 (17.3)	8 (13.6)
T2D mellitus	2,081 (31.9)	586 (32.5)	938 (28.8)	523 (37.4)	34 (57.6)
Hypertension	4,538 (69.6)	1,022 (56.7)	2,297 (70.4)	1,167 (83.4)	52 (88.1)
Peripheral artery disease	352 (5.4)	99 (5.5)	166 (5.1)	81 (5.8)	6 (10.2)
AKI before randomization	201 (3.1)	19 (1.1)	47 (1.4)	109 (7.8)	26 (44.1)
eGFR (ml min^−1^ 1.73 m^−^^2^), mean (s.d.)	76.12 (19.94)	99.32 (7.59)	75.88 (8.69)	48.94 (7.86)	25.53 (3.68)
Body mass index (kg m^−^^2^), mean (s.d.)^a^	28.07 (5.02)	27.84 (5.09)	27.96 (4.91)	28.54 (5.10)	29.74 (6.06)
SBP (mmHg), mean (s.d.)^a^	120.4 (14.9)	119.1 (14.8)	120.5 (14.7)	121.9 (15.0)	123.1 (16.5)
DBP (mmHg), mean (s.d.)^a^	73.4 (10.0)	73.7 (10.0)	73.5 (10.0)	73.0 (9.7)	71.3 (11.7)
NT-proBNP (pg ml^−1^), median (IQR)^b^	1,825.21 (741.30–3,624.00)	1,540.00 (662.00–2,642.00)	1,784.50 (680.50–3,480.00)	2,790.00 (1,164.00–6,309.00)	6,280.00 (3,635.00–11,599.00)
Medication at baseline					
ACEi/ARB/ARNi	4,726 (72.5)	1,309 (72.6)	2,386 (73.2)	1,002 (71.6)	29 (49.2)
MRA	2,573 (39.5)	663 (36.8)	1,296 (39.7)	597 (42.7)	17 (28.8)
Loop diuretics	2,212 (33.9)	497 (27.6)	1,041 (31.9)	635 (45.4)	39 (66.1)
Medication at discharge					
ACEi/ARB/ARNi	5,381 (82.5)	1,496 (83.0)	2,710 (83.1)	1,142 (81.6)	33 (55.9)
MRA	3,117 (47.8)	826 (45.8)	1,583 (48.5)	690 (49.3)	18 (30.5)
Loop diuretics	2,499 (38.3)	556 (30.8)	1,184 (36.3)	719 (51.4)	40 (67.8)

Data are *n* (%) unless stated otherwise. Demographics, medical conditions and other baseline characteristics among patients randomized to empagliflozin or placebo in EMPACT-MI. Patients were stratified according to baseline kidney function and according to the following eGFR categories: <30 ml min^−1^ 1.73 m^−^^2^; ≥30 to <60 ml min^−1^ 1.73 m^−^^2^; ≥60 to <90 ml min^−1^ 1.73 m^−^^2^; and ≥90 ml min^−1^ 1.73 m^−^^2^. Kidney function was determined according to the eGFR using the CKD-EPI equation. LVEF, left ventricular ejection fraction; DBP, diastolic blood pressure; T2D, type 2 diabetes. ^a^Number of patients in the overall population with missing data for index MI type: *n* = 2; lowest LVEF: *n* = 52; baseline body mass index: *n* = 27; baseline SBP: *n* = 1; baseline DBP: *n* = 1. ^b^Collection not mandatory; based on 2,548 patients overall, 759 patients with an eGFR ≥ 90 ml min^−1^ 1.73 m^−^^2^, 1,280 patients with an eGFR ≥60 to <90 ml min^−1^ 1.73 m^−^^2^, 486 patients with an eGFR ≥30 to <60 ml min^−1^ 1.73 m^−^^2^ and 23 patients with an eGFR <30 ml min^−1^ 1.73 m^−^^2^.

There were 1,267 (19.4%) patients randomized in Bulgaria, Germany, Hungary and Serbia, with 1,171 patients providing eGFR data, of which a subset of 1,152 (17.7%) patients were treated with the study drug and provided eGFR data at baseline and at least at one visit after baseline during the on-treatment time. Among this subset of patients, a total of 564 (49.0%) were randomized to empagliflozin and 588 (51.0%) to placebo. A total of 236 (20.5%) of these patients provided data at the 24-month time point. The baseline characteristics of this subgroup are shown in Supplementary Table 2.

### Relevant therapy initiation at index hospitalization

At the time of randomization, a total of 4,726 (72.5%) patients were on angiotensin-converting enzyme inhibitor (ACEi)/angiotensin receptor blocker (ARB)/angiotensin receptor/neprilysin inhibitor (ARNi) therapy, 2,573 (39.5%) on a mineralocorticoid receptor antagonist (MRA) and 2,212 (33.9%) on loop diuretics. By discharge, more patients were receiving ACEi/ARB/ARNi therapy (82.5%), MRAs (47.8%) and loop diuretics (38.3%) (Table 1). During the index hospitalization, there was a similar proportion of patients in the empagliflozin and placebo groups who were newly initiated on ACEi/ARB/ARNi therapy (59.8% and 58.6%), MRAs (44.4% and 44.0%) and loop diuretics (36.3% and 33.0%) (Table 2).

**Table 2:** Medical therapy use at index hospitalization

	ACEi/ARB/ARNi		MRA		Loop diuretics	
	Empagliflozin (*n* = 3,260)	Placebo (*n* = 3,262)	Empagliflozin (*n* = 3,260)	Placebo (*n* = 3,262)	Empagliflozin (*n* = 3,260)	Placebo (*n* = 3,262)
Not receiving the medication at discharge	577 (17.7)	564 (17.3)	1,718 (52.7)	1,687 (51.7%)	1,959 (60.1)	2,064 (63.3)
Not on medication at discharge and no intake before discharge	511 (15.7)	508 (15.6)	1,671 (51.3)	1,642 (50.3)	1,704 (52.3)	1,777 (54.5)
Patients newly initiating treatment during the index hospitalization and stopped the treatment before discharge	66 (2.0)	56 (1.7)	47 (1.4)	45 (1.4)	255 (7.8)	287 (8.8)
Receiving the medication at discharge	2,683 (82.3)	2,698 (82.7)	1,542 (47.3)	1,575 (48.3)	1,301 (39.9)	1,198 (36.7)
Medication initiated before the index hospitalization and on the medication at discharge	735 (22.5)	785 (24.1)	96 (2.9)	139 (4.3)	118 (3.6)	121 (3.7)
New initiation during the index hospitalization and on the medication at discharge	1,948 (59.8)	1,913 (58.6)	1,446 (44.4)	1,436 (44.0)	1,183 (36.3)	1,077 (33.0)

Data are *n* (%). Key additional medical therapy use among patients in EMPACT-MI randomized to empagliflozin or placebo. Data are demonstrated at, during or on discharge from the index hospitalization for acute MI for ACEi/ARB/ARNi, MRA and loop diuretic medical therapies.

### Kidney outcomes

**Fig. 1:**
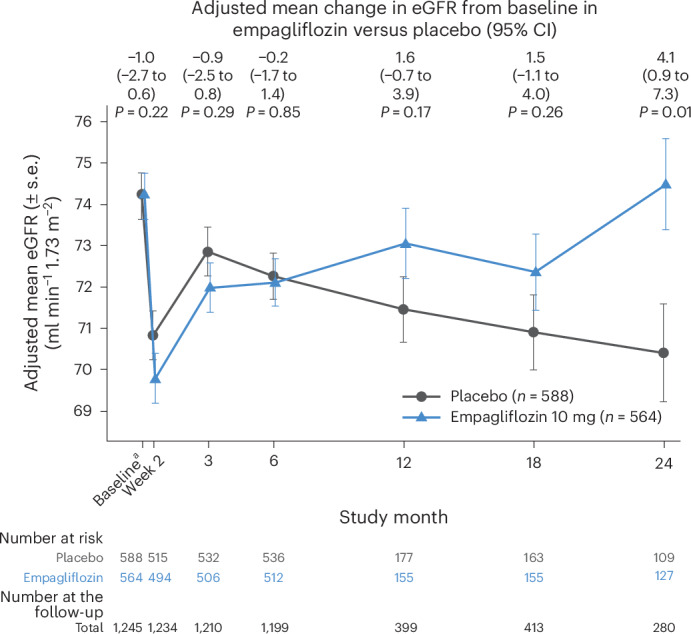
Change in kidney function. Patients randomized to empagliflozin or placebo with longitudinal eGFR data in EMPACT-MI. Longitudinal eGFR data were obtained only for a subset of patients, specifically those treated with the study drug, in Bulgaria, Germany, Hungary and Serbia, and with available data (*n* = 1,152, 17.7%). Patients were compared overall. The eGFR was determined using the Chronic Kidney Disease Epidemiology Collaboration (CKD-EPI) equation. Changes in eGFR over time on treatment were assessed using mixed models to account for repeated eGFR values. Models included age as a linear covariate and sex, geographical region, baseline LVEF, T2D status, persistent or permanent atrial fibrillation, previous MI, peripheral artery disease, smoking status, baseline eGFR according to visit, and visit according to treatment interactions as fixed effects. All significance testing was two-sided without adjustment for multiple testing. Estimates are shown with the 95% CI bars. The number of patients at the follow-up are presented for pooled treatment groups. The number of patients at the follow-up at 12 months excludes all patients counted at the 18-month and 24-month visits because only the latest of the 12–24-month measurements was expected. The number of patients at the follow-up at 18 months excludes all patients counted at the 24-month visits because only the latest of the 12–24-month measurements was expected. ^a^Unadjusted baseline pooled over both treatment groups.

**Extended Data Fig. 1:**
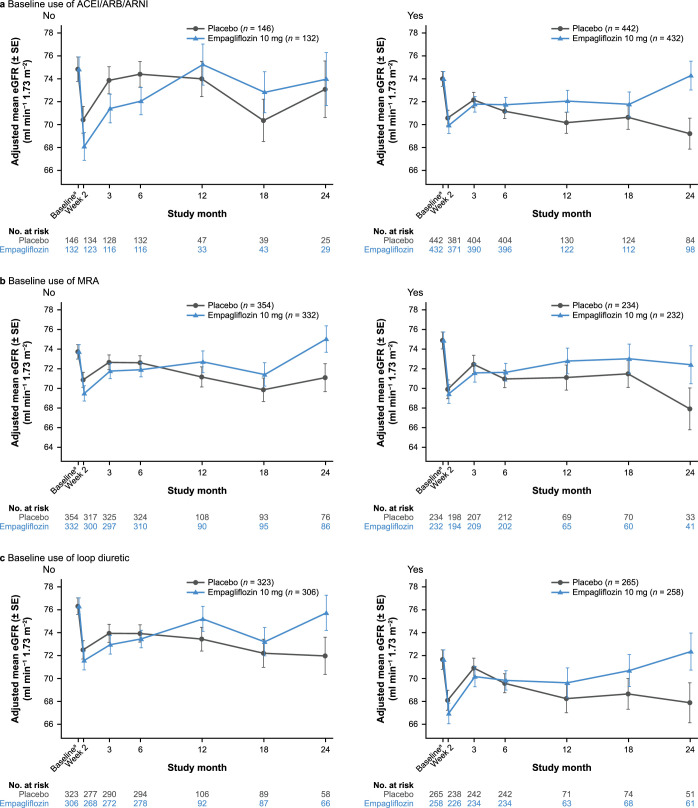
Change in Kidney Function by Baseline Use of Additional Medical Therapies. Patients randomized to empagliflozin or placebo with longitudinal eGFR data were compared in EMPACT-MI from baseline to 24 months. Patients were stratified by baseline use or not use of key therapies, specifically ACEi/ARB/ARNi (panel **a**), MRA (panel **b**), and loop diuretics (panel **c**). Longitudinal eGFR data was obtained only for a subset of patients, specifically those treated in Bulgaria, Germany, Hungary, and Serbia. Changes in eGFR over time on-treatment was assessed using mixed models to account for repeated eGFR values. Models that age as a linear covariate and sex, geographic region, baseline left ventricular ejection fraction, type 2 diabetes status, persistent or permanent atrial fibrillation, previous myocardial infarction, peripheral artery disease, smoking status, baseline estimated GFR according to visit, visit according to treatment interactions as fixed effects and subgroup according to visit according to treatment interaction. Estimates are shown with 95% confidence intervals bars. Abbreviations: ACEi, angiotensin-converting enzyme; AE, adverse events; ARB, angiotensin II receptor blockers; ARNI, angiotensin receptor/neprilysin inhibitor; HF, eGFR, estimated glomerular filtration rate; heart failure; HHF, heart failure hospitalization, MRA, mineralocorticoid receptor antagonist.

Among patients in the longitudinal eGFR subset, mean age was 62.4 years (s.d. = 11.0 years) and 63.8 years (s.d. = 11.1 years) and baseline eGFR was 75.3 ml min^−1^ 1.73 m^−^^2^ (s.e. = 0.8 ml min^−1^ 1.73 m^−^^2^) and 73.1 ml min^−1^ 1.73 m^−^^2^ (s.e. = 0.8 ml min^−1^ 1.73 m^−^^2^) in the empagliflozin and placebo groups, respectively. Two weeks from randomization, there was a similar initial decline in eGFR with an adjusted mean eGFR change of −4.8 ml min^−1^ 1.73 m^−^^2^ (95% confidence interval CI = −5.9 to −3.6 ml min^−1^ 1.73 m^−^^2^) in the empagliflozin group and of −3.7 ml min^−1^ 1.73 m^−^^2^ (95% CI = −4.9 to −2.6 ml min^−1^ 1.73 m^−^^2^) in the placebo group (adjusted mean difference = −1.0, 95% CI = −2.7 to 0.6; *P* = 0.22) (Fig. 1). Notably, the initial decline in eGFR observed at 2 weeks was comparable between empagliflozin and placebo among patients who were treated using ACEi/ARB/ARNi therapy, MRAs or loop diuretics at baseline (Extended Data Fig. 1).

**Fig. 2:**
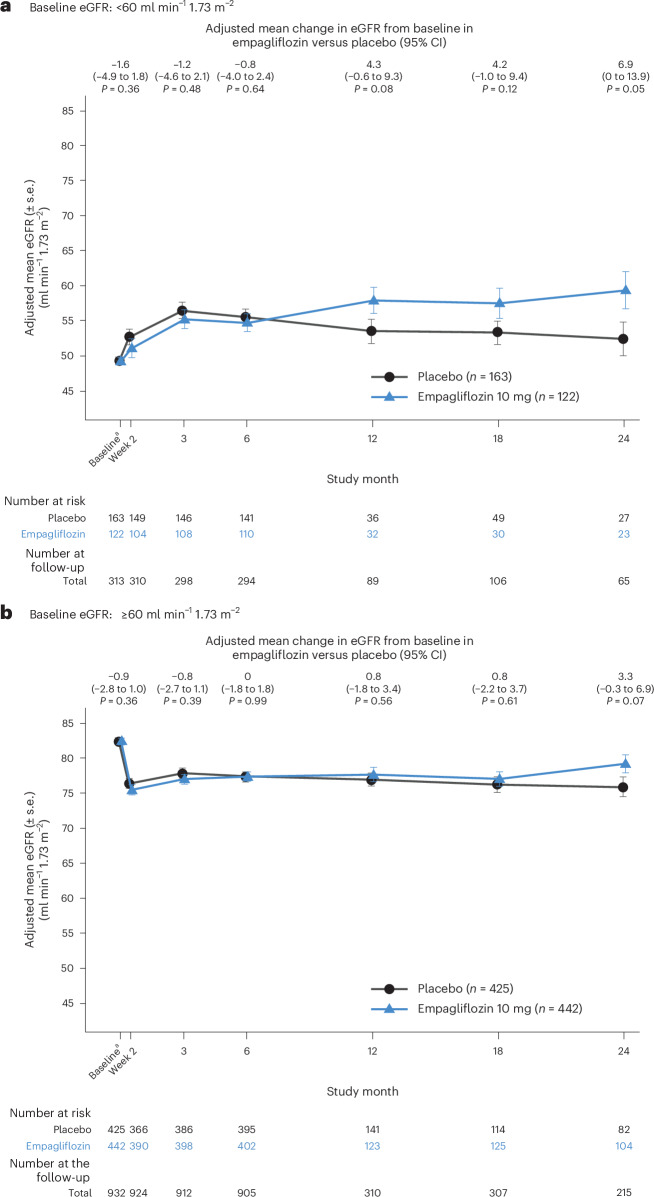
Change in kidney function according to baseline kidney function. **a**,**b**, Patients randomized to empagliflozin or placebo with longitudinal eGFR data in EMPACT-MI. Longitudinal eGFR data were obtained only for a subset of patients, specifically those treated with the study drug, in Bulgaria, Germany, Hungary and Serbia, and with available data (*n* = 1,152, 17.7%). Patients were compared according to a baseline kidney function of <60 ml min^−1^ 1.73 m^−^^2^ (**a**) and ≥60 ml min^−1^ 1.73 m^−^^2^ (**b**). The eGFR was determined using the CKD-EPI equation. Changes in eGFR over time on treatment were assessed using mixed models to account for repeated eGFR values. Models included age as a linear covariate and sex, geographical region, baseline LVEF, T2D status, persistent or permanent atrial fibrillation, previous MI, peripheral artery disease, smoking status, baseline eGFR according to visit and visit according to treatment interactions as fixed effects. The model for the subgroup analyses included an additional factor for the subgroup according to visit and according to treatment interaction. All significance testing was two-sided without adjustment for multiple testing. Estimates are shown with the 95% CI bars. The number of patients at the follow-up are presented for the pooled treatment groups. The number of patients at the 12-month follow-up excludes all patients counted at the 18-month and 24-month visits because only the latest of the 12–24-month measurements was expected. The number of patients at the 18-month follow-up excludes all patients counted at the 24-month visits because only the latest of the 12–24-month measurements was expected. ^a^Unadjusted baseline pooled over both treatment groups.

**Extended Data Fig. 2:**
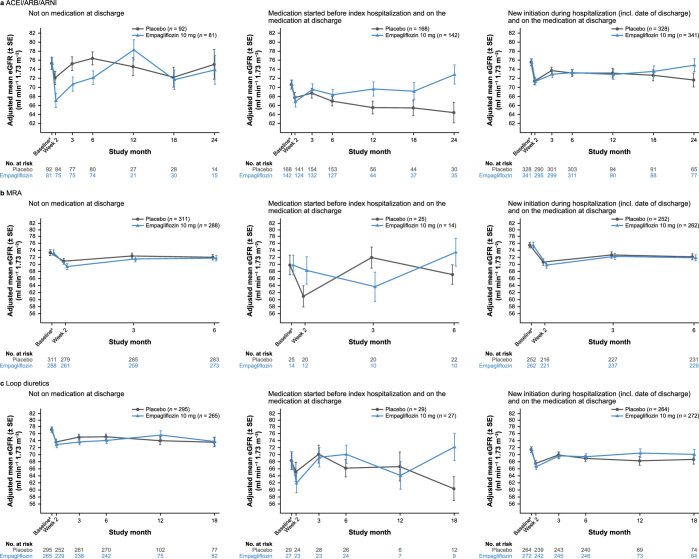
Change in Kidney Function by Use of Additional Medical Therapies. Patients randomized to empagliflozin or placebo with longitudinal eGFR data were compared in EMPACT-MI from baseline to 24 months. Patients were stratified by use key therapies, specifically ACEi/ARB/ARNi (panel **a**), MRA (panel **b**), and loop diuretics (panel **c**) at discharge with introduction prior to index hospitalization, at discharge with introduction during index hospitalization and not on medication at discharge. Longitudinal eGFR data was obtained only for a subset of patients, specifically those treated in Bulgaria, Germany, Hungary, and Serbia. Changes in eGFR over time on-treatment was assessed using mixed models to account for repeated eGFR values. Models that age as a linear covariate and sex, geographic region, baseline left ventricular ejection fraction, type 2 diabetes status, persistent or permanent atrial fibrillation, previous myocardial infarction, peripheral artery disease, smoking status, baseline estimated GFR according to visit, visit according to treatment interactions as fixed effects and subgroup according to visit according to treatment interaction. Estimates are shown with 95% confidence intervals bars. Abbreviations: ACEi, angiotensin-converting enzyme; AE, adverse events; ARB, angiotensin II receptor blockers; ARNI, angiotensin receptor/neprilysin inhibitor; HF, eGFR, estimated glomerular filtration rate; heart failure; HHF, heart failure hospitalization, MRA, mineralocorticoid receptor antagonist.

Subsequently, by 3 months, there was a mean improvement in eGFR in both groups, although after this time the eGFR improved further in the empagliflozin group but declined in the placebo group (Fig. 1). By 24 months, patients in the empagliflozin group had an eGFR similar to baseline (adjusted mean eGFR change: −0.1 ml min^−1^ 1.73 m^−^^2^; 95% CI = −2.2, 2.1 ml min^−1^ 1.73 m^−^^2^), while patients in the placebo group had a decrease in eGFR from baseline (adjusted mean eGFR change = −4.1 ml min^−1^ 1.73 m^−^^2^, 95% CI = −6.5 to −1.8 ml min^−1^ 1.73 m^−^^2^). The adjusted mean difference in eGFR change between empagliflozin and placebo was 4.1 ml min^−1^ 1.73 m^−^^2^ (95% CI = 0.9 to 7.3 ml min^−1^ 1.73 m^−^^2^; *P* = 0.01), indicating lower eGFR in the placebo group by month 24 (Fig. 1). The findings were consistent with a baseline eGFR of <60 ml min^−1^ 1.73 m^−^^2^ or ≥60 ml min^−1^ 1.73 m^−^^2^ (*P*_interaction_ = 0.36) (Fig. 2) and baseline use of ACEi/ARB/ARNi therapy (*P*_interaction_ = 0.28), MRA (*P*_interaction_ = 0.87) or loop diuretics (*P*_interaction_ = 0.83) (Extended Data Fig. 1). Similar results were observed for patients who had newly initiated ACEi/ARB/ARNi therapy, MRA or loop diuretics during the index hospitalization (Extended Data Fig. 2).

**Extended Data Fig. 3:**
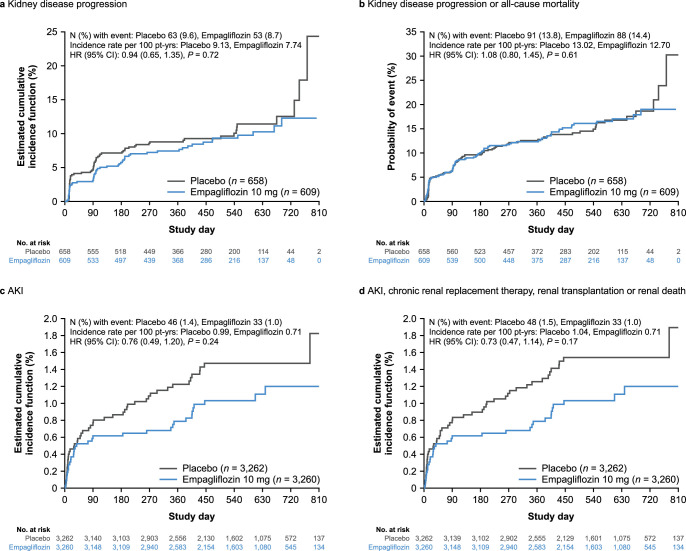
Cumulative Incidence of Kidney Outcomes. Patients randomized to empagliflozin or placebo were compared in EMPACT-MI. The endpoints included the following time-to-first event endpoints: kidney disease progression (panel **a**); kidney disease progression or all-cause mortality (panel **b**); AKI (panel **c**); and AKI, chronic renal replacement therapy, renal transplantation, or death (panel **d**). The x-axis shows time from randomization in days and the y-axis shows estimated cumulative incidence function or respectively Kaplan-Meier estimates (panel **b**). First kidney disease progression was defined as time to first decline of eGFR ≥40% from baseline, estimated GFR < 15 ml min^−1^ 1.73 m^−2^ for patients with baseline estimated GFR ≥ 30 ml min^−1^ 1.73 m^−2^ or <10 ml min^−1^ 1.73 m^−2^ for patients with baseline eGFR <30 ml min^−1^ 1.73 m^−2^. Renal death was defined as a death event with primary adverse event leading to death in narrow Sandardized MedDRA queries (SMQ),’Acute Renal Failure’. Chronic renal replacement therapy was defined as renal replacement therapy lasting for at least 90 days. Differences between the empagliflozin and placebo group were assessed with Cox proportional hazard models. A HR less than 1 indicates reduction in risk for the endpoint with empagliflozin while a HR of greater than 1 indicating increase in risk for the endpoint with empagliflozin. Models included adjustment for the baseline covariates of age, sex, geographic region, eGFR, left ventricular ejection fraction ( < 35% or ≥35%), type 2 diabetes status, persistent or permanent atrial fibrillation, previous myocardial infarction, peripheral artery disease, and smoking status. All significance testing was two-sided without adjustment for multiple testing. Abbreviations: AKI, acute kidney injury; eGFR, estimated glomerular filtration rate; HR, hazard ratio.

Patients in the empagliflozin group, compared with the placebo group, had a numerically lower rate of the composite outcome of AKI, chronic renal replacement therapy, renal transplantation or renal death (0.71 versus 1.04 patients with event per 100 person years; hazard ratio (HR) = 0.73, 95% CI = 0.47 to 1.14, *P* = 0.17) but this was not statistically significant. A similar pattern was observed for AKI (0.71 versus 0.99 patients with event per 100 person years; HR = 0.76, 95% CI = 0.49 to 1.20, *P* = 0.24; Extended Data Fig. 3). Among patients in the longitudinal eGFR subgroup, kidney disease progression rates were similar (*P* = 0.72), as was the composite of kidney disease progression or all-cause mortality (*P* = 0.61) (Extended Data Fig. 3).

**Extended Data Fig. 4:**
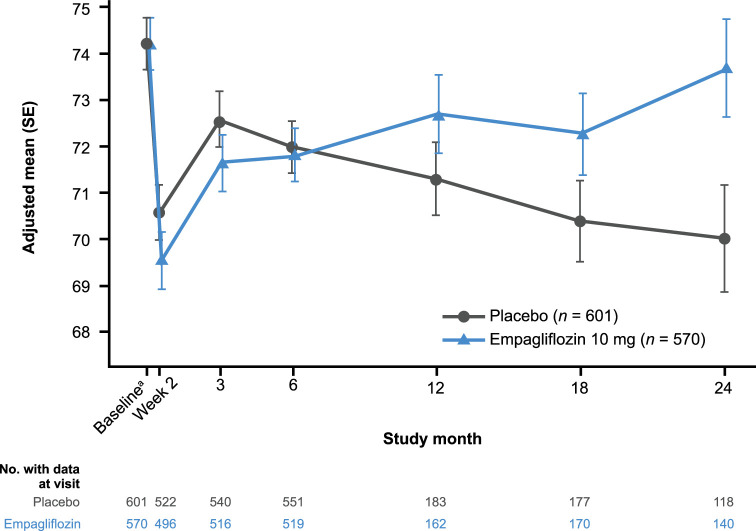
Change in Kidney Function Over Time During the Study. Patients randomized to empagliflozin or placebo with longitudinal eGFR data in EMPACT-MI. Longitudinal eGFR data was obtained only for a subset of patients, specifically those randomised in Bulgaria, Germany, Hungary, and Serbia. Analysis is through end of study and not restricted to time on study drug. Changes in eGFR over time was assessed using mixed models to account for repeated eGFR values. Changes in eGFR over time was assessed using mixed models to account for repeated eGFR values. Models included age as a linear covariate and sex, geographic region, baseline left ventricular ejection fraction, type 2 diabetes status, persistent or permanent atrial fibrillation, previous myocardial infarction, peripheral artery disease, smoking status, baseline estimated GFR according to visit, and visit according to treatment interactions as fixed effects. Estimates are shown with 95% confidence interval bars.

Results for changes in eGFR over time on treatment (Fig. 1) were consistent with changes in eGFR over time during the study (Extended Data Fig. 4). Results for changes in eGFR to last value on treatment and to last value in study showed a larger decline in the placebo arm compared to empagliflozin (Supplementary Tables 3 and 4).

### HF outcomes

**Fig. 3:**
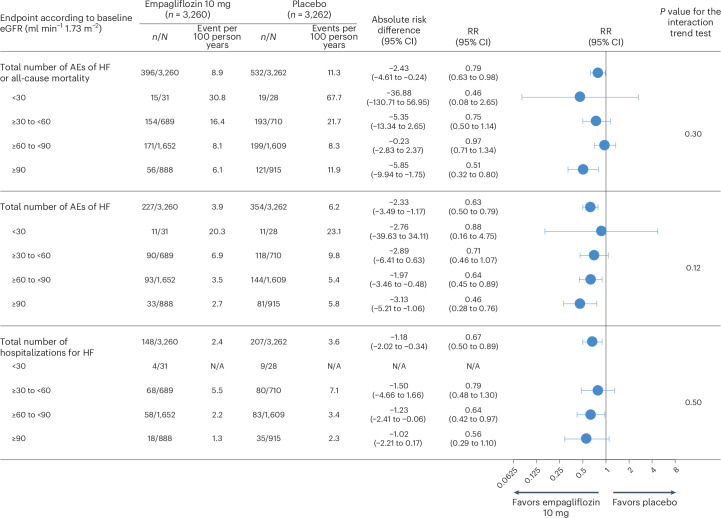
HF endpoints according to baseline kidney function. Patients randomized to empagliflozin or placebo in EMPACT-MI. Patients were stratified according to baseline kidney function to the eGFR categories of <30 ml min^−1^ 1.73 m^−^^2^, ≥30 to <60 ml min^−1^ 1.73 m^−^^2^, ≥60 to <90 ml min^−1^ 1.73 m^−^^2^ and ≥90 ml min^−1^ 1.73 m^−^^2^. The absolute risk difference represents the adjusted rate difference per 100 patient years at risk. Endpoints included a composite of the total number of AEs of HF or all-cause mortality, the total number of AEs of HF and the total number of hospitalizations for HF. The AEs of HF were defined as investigator-reported AEs categorized as ‘cardiac failure’ according to the Medical Dictionary for Regulatory Activities (MedDRA), and included not only the events analyzed as the prespecified endpoint of HF hospitalization but a broader range of AEs of HF, including outpatient non-fatal AEs and those requiring or prolonging hospitalization or with a fatal outcome. Kidney function was determined as the eGFR using the CKD-EPI equation. Differences in HF outcomes between the empagliflozin and placebo groups were assessed using negative binomial regression models and included total events (first and recurrent). An interaction term for treatment and baseline eGFR category was included in the models. Models included adjustment for the baseline covariates of age, sex, geographical region, eGFR, LVEF (<35% or ≥35%), T2D status, persistent or permanent atrial fibrillation, previous MI, peripheral artery disease and smoking status. All significance testing was two-sided without adjustment for multiple testing. The figure shows the RR with the 95% CI error bars. N/A, not applicable.

**Extended Data Fig. 5:**
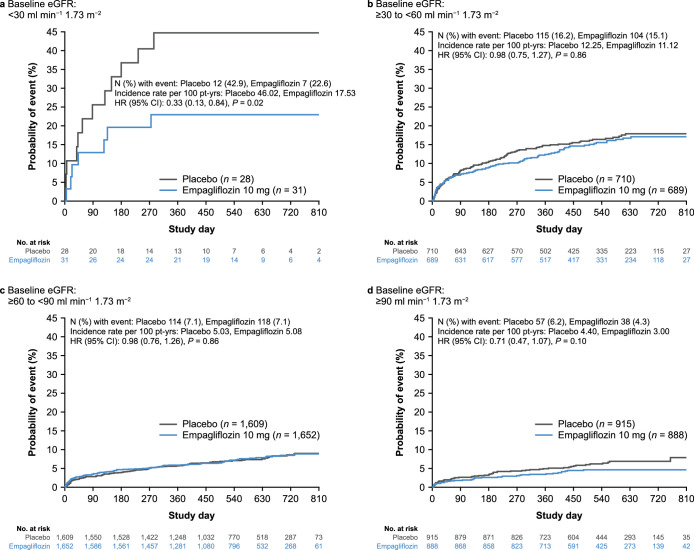
Kaplan Meier Estimates of Heart Failure Hospitalization or All-Cause Mortality by Kidney Function. Patients randomized to empagliflozin or placebo were compared in EMPACT-MI. The endpoint was a composite of time to first HHF or all-cause mortality. Patients were stratified by baseline kidney function, to categories eGFR of <30 ml min^−1^ 1.73 m^−2^ (panel **a**), 30- < 60 ml min^−1^ 1.73 m^−2^ (panel **b**), 60- < 90 ml min^−1^ 1.73 m^−2^ (panel **c**), ≥90 ml min^−1^ 1.73 m^−2^ (panel **d**). Kidney function was determined by eGFR with the CKD-EPI equation. The x-axis shows time from randomization in days and the y-axis shows probability of event based on Kaplan Meier estimates. Differences between the empagliflozin and placebo group were assessed with a Cox proportional hazard model. A HR less than 1 indicates reduction in risk for the endpoint with empagliflozin while a HR of greater than 1 indicates an increase in risk for the endpoint with empagliflozin. An interaction term for treatment and baseline eGFR category was included. The models included adjustment for the baseline covariates of age, sex, geographic region, eGFR, left ventricular ejection fraction ( < 35% or ≥35%), type 2 diabetes status, persistent or permanent atrial fibrillation, previous myocardial infarction, peripheral artery disease, and smoking status. All significance testing was two-sided without adjustment for multiple testing. No significant interaction was observed between treatment effect and baseline kidney function (Pinteraction = 0.81). Abbreviations: CI, confidence interval; eGFR, estimated glomerular filtration rate; HR, hazard ratio; HHF, heart failure hospitalization.

The composite outcome of hospitalization for HF or all-cause mortality had a similar rate among the empagliflozin and placebo groups (HR = 0.90, 95% CI = 0.76 to 1.06, *P* = 0.21), with a numerically lower rate in the empagliflozin group, although these findings were not statistically significant; however, they were consistent across baseline eGFR (Extended Data Fig. 5). Empagliflozin reduced total hospitalization for HF (Fig. 3), with 118 events (2.4 events per 100 person years) in the empagliflozin group and 153 events (3.6 events per 100 person years) in the placebo group (rate ratio (RR) = 0.67, 95% CI = 0.50 to 0.89, *P* = 0.006), with consistent effects according to kidney function (*P*_interaction_ = 0.50).

**Fig. 4:**
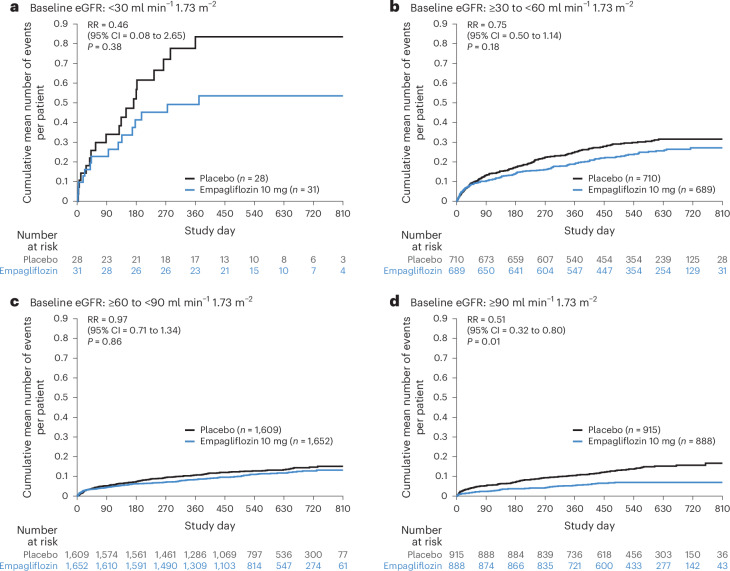
Total AEs of HF or all-cause mortality according to kidney function (four categories). **a**–**d**, Patients randomized to empagliflozin or placebo in EMPACT-MI. The endpoint was a composite of the total number of AEs of HF or all-cause mortality. Patients were stratified according to baseline kidney function to the eGFR categories of <30 ml min^−1^/1.73 m^−^^2^ (**a**), ≥30 to <60 ml min^−1^ 1.73 m^−^^2^ (**b**), ≥60 to <90 ml min^−1^ 1.73 m^−^^2^ (**c**) and ≥90 ml min^−1^ 1.73 m^−^^2^ (**d**). Kidney function was determined according to the eGFR using the CKD-EPI equation. The AEs of HF were defined as investigator-reported AEs that were categorized as ‘cardiac failure’ according to MedDRA, and which included not only the events analyzed as the prespecified endpoint of HF hospitalization but also the broader range of AEs of HF, including outpatient non-fatal AEs and those requiring or prolonging hospitalization or with a fatal outcome. The *x* axis shows the time from randomization in days and the *y* axis shows the cumulative mean number of events per patient. Differences in endpoint between the empagliflozin and placebo group were assessed using a negative binomial regression model and included total events (first and recurrent). An interaction term for treatment and baseline eGFR category was included. The model included adjustment for the baseline covariates of age, sex, geographical region, eGFR, LVEF (<35% or ≥35%), T2D status, persistent or permanent atrial fibrillation, previous MI, peripheral artery disease and smoking status. All significance testing was two-sided without adjustment for multiple testing. No significant interaction was observed between treatment and baseline kidney function (*P*_interaction_ = 0.30).

**Extended Data Fig. 6:**
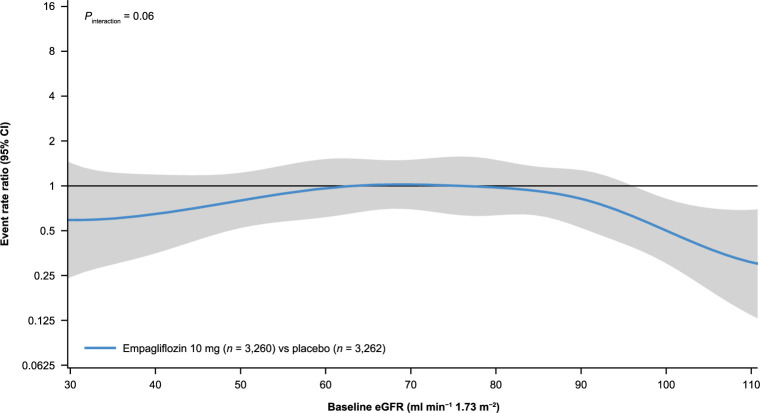
Total Adverse Events of HF or All-Cause Mortality by Continuous Kidney Function. Patients randomized to empagliflozin or placebo in EMPACT-MI. The endpoint was a composite of total number of AEs of HF or all-cause mortality. Baseline kidney function was evaluated continuously by eGFR. Kidney function was determined by eGFR with the CKD-EPI equation. The adverse events of Heart Failure were defined as investigator-reported adverse events that were categorized as “cardiac failure” per MedDRA,21 and included not only the events analyzed as prespecified endpoint of heart failure hospitalization but broader range of adverse events of heart failure including outpatient non-fatal adverse events as well as those requiring or prolonging hospitalization or with a fatal outcome. The x-axis shows baseline eGFR and the y-axis shows event rate ratio for empagliflozin compared with placebo. The shaded area shows the 95% Confidence interval. A rate ratio less than 1 indicates reduction in the risk of outcome with empagliflozin while a rate ratio of greater than 1 indicates an increase in the risk of outcome with empagliflozin. Differences between the empagliflozin and placebo group were assessed across the baseline eGFR range using cubic splines. The negative binomial regression model included adjustment for the baseline covariates of age, sex, geographic region, eGFR, left ventricular ejection fraction ( < 35% or ≥35%), type 2 diabetes status, persistent or permanent atrial fibrillation, previous myocardial infarction, peripheral artery disease, smoking status and eGFR-by-treatment interaction. All significance testing was two-sided without adjustment for multiple testing. No significant interaction was observed between treatment effect and baseline kidney function (Pinteraction = 0.06). Abbreviations: AE, adverse events; CI, confidence interval; eGFR, estimated glomerular filtration rate; HF, heart failure.

Patients randomized to empagliflozin had a lower rate of adverse events (AEs) of HF or all-cause mortality events (396 events, 8.9 events per 100 person years) compared with placebo (532 events, 11.3 events per 100 person years; RR = 079, 95% CI = 0.63 to 0.98, *P* = 0.03). Empagliflozin consistently decreased the total AEs of HF or all-cause mortality irrespective of baseline eGFR group, including eGFR ≥90 ml min^−1^ 1.73 m^−^^2^ (RR = 0.51, 95% CI = 0.32 to 0.80), ≥60 to <90 ml min^−1^ 1.73 m^−^^2^ (RR = 0.97, 95% CI = 0.71 to 1.34), ≥30 to <60 ml min^−1^ 1.73 m^−^^2^ (RR = 0.75, 95% CI = 0.50 to 1.14) and <30 ml min^−1^ 1.73 m^−^^2^ (RR = 0.46, 95% CI = 0.08 to 2.65), without significant interaction of treatment and baseline eGFR group (*P*_interaction_ = 0.30) (Figs. 3 and 4 and Extended Data Fig. 6).

**Extended Data Fig. 7:**
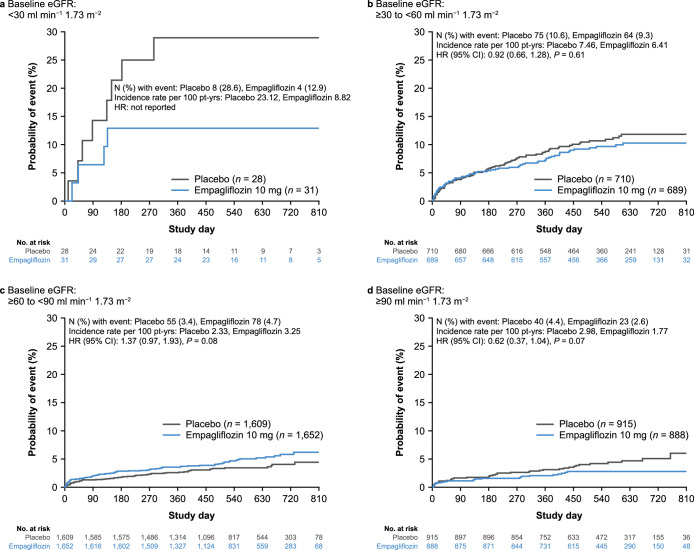
Kaplan Meier Estimates of All-Cause Mortality by Kidney Function. Patients randomized to empagliflozin or placebo were compared in EMPACT-MI. The endpoint was all-cause mortality. Patients were stratified by baseline kidney function, to categories eGFR of <30 ml min^−1^ 1.73 m^−2^ (panel **a**), 30- < 60 ml min^−1^ 1.73 m^−2^ (panel **b**), 60- < 90 ml min^−1^ 1.73 m^−2^ (panel **c**), ≥90 ml min^−1^ 1.73 m^−2^ (panel **d**). Kidney function was determined by eGFR with the CKD-EPI equation. The x-axis shows time from randomization in days and the y-axis shows probability of event based on Kaplan Meier estimates. Differences between the empagliflozin and placebo group were assessed with Cox proportional hazard models. A HR less than 1 indicates reduction in risk for the endpoint with empagliflozin while a HR of greater than 1 indicates increased risk in endpoint with empagliflozin. An interaction term for treatment and baseline eGFR category was included. The model included adjustment for the baseline covariates of age, sex, geographic region, eGFR, left ventricular ejection fraction ( < 35% or ≥35%), type 2 diabetes status, persistent or permanent atrial fibrillation, previous myocardial infarction, peripheral artery disease, and smoking status. HR was not reported for the eGFR of <30 ml min^−1^ 1.73 m^−2^ group due to less than 14 patients with events. All significance testing was two-sided without adjustment for multiple testing. No significant interaction was observed between treatment effect and baseline kidney function (Pinteraction = 0.89). Abbreviations: CI, confidence interval; eGFR, estimated glomerular filtration rate; HR, hazard ratio.

This observed reduction was primarily driven by a decrease in the AEs of HF (227 versus 354 events, 3.9 versus 6.2 events per 100 person years; RR = 0.63, 95% CI = 0.50 to 0.79, *P* < 0.0001; Fig. 3), as reduction in all-cause mortality was not observed (169 versus 178 events, 3.6 versus 3.8 events per 100 person years) (HR = 0.96, 95% CI = 0.78 to 1.19, *P* = 0.73). Both these endpoints had consistent findings according to baseline eGFR group (*P*_interaction_ = 0.12 and *P*_interaction_ = 0.89, respectively) (Fig. 3 and Extended Data Fig. 7).

### AEs

A similar rate of any AEs within 30 days after the first study drug intake was observed in the empagliflozin (10.2%) and placebo (10.1%) groups. This was similar according to baseline eGFR ≥60 ml min^−1^ 1.73 m^−^^2^ (9.5% versus 8.7%) and <60 ml min^−1^ 1.73 m^−^^2^ (13.0% versus 14.9%), baseline systolic blood pressure (SBP) < 110 mmHg (13.0% versus 14.1%), 110 ≤ 130 mmHg (9.4% versus 10.0%) and ≥130 mmHg (9.5% versus 7.4%), baseline ACEi/ARB/ARNi use (9.8% versus 9.6%), baseline MRA use (11.0% versus 9.9%) and baseline loop diuretic use (12.6% versus 12.2%) (Table 3).

**Table 3:** AEs within 30 days after the first dose of the study drug

Category at baseline		Any AE		AE leading to treatment discontinuation		Serious AE		Acute renal failure^a^		Contrast-induced AKI		Volume depletion^b^		Hypotension^b^	
		Placebo	Empagliflozin	Placebo	Empagliflozin	Placebo	Empagliflozin	Placebo	Empagliflozin	Placebo	Empagliflozin	Placebo	Empagliflozin	Placebo	Empagliflozin
		*n*/*N* (%)	*n*/*N* (%)	*n*/*N* (%)	*n*/*N* (%)	*n*/*N* (%)	*n*/*N* (%)	*n*/*N* (%)	*n*/*N* (%)	*n*/*N* (%)	*n*/*N* (%)	*n*/*N* (%)	*n*/*N* (%)	*n*/*N* (%)	*n*/*N* (%)
Overall		327/3,229 (10.1)	331/3,234 (10.2)	42/3,229 (1.3)	51/3,234 (1.6)	274/3,229 (8.5)	269/3,234 (8.3)	27/3,229 (0.8)	22/3,234 (0.7)	9/3,229 (0.3)	6/3,234 (0.2)	11/3,229 (0.3)	17/3,234 (0.5)	10/3,229 (0.3)	17/3,234 (0.5)
eGFR, ml min^−1^ 1.73 m^−^^2^	<60	109/732 (14.9)	92/710 (13.0)	17/732 (2.3)	14/710 (2.0)	94/732 (12.8)	80/710 (11.3)	19/732 (2.6)	15/710 (2.1)	7/732 (1.0)	3/710 (0.4)	3/732 (0.4)	3/710 (0.4)	2/732 (0.3)	3/710 (0.4)
	≥60	218/2,497 (8.7)	239/2,524 (9.5)	25/2,497 (1.0)	37/2,524 (1.5)	180/2,497 (7.2)	189/2,524 (7.5)	8/2,497 (0.3)	7/2,524 (0.3)	2/2,497 (0.1)	3/2,524 (0.1)	8/2,497 (0.3)	14/2,524 (0.6)	8/2,497 (0.3)	14/2,524 (0.6)
SBP (mmHg)	<110	101/714 (14.1)	93/713 (13.0)	12/714 (1.7)	12/713 (1.7)	89/714 (12.5)	80/713 (11.2)	9/714 (1.3)	5/713 (0.7)	2/714 (0.3)	1/713 (0.1)	3/714 (0.4)	5/713 (0.7)	3/714 (0.4)	5/713 (0.7)
	110 to <130	155/1,555 (10.0)	150/1,594 (9.4)	20/1,555 (1.3)	23/1,594 (1.4)	131/1,555 (8.4)	115/1,594 (7.2)	11/1,555 (0.7)	7/1,594 (0.4)	5/1,555 (0.3)	2/1,594 (0.1)	6/1,555 (0.4)	7/1,594 (0.4)	6/1,555 (0.4)	7/1,594 (0.4)
	≥130	71/960 (7.4)	88/926 (9.5)	10/960 (1.0)	16/926 (1.7)	54/960 (5.6)	74/926 (8.0)	7/960 (0.7)	10/926 (1.1)	2/960 (0.2)	3/926 (0.3)	2/960 (0.2)	5/926 (0.5)	1/960 (0.1)	5/926 (0.5)
ACEi/ARB/ARNi	Yes	222/2,310 (9.6)	233/2,376 (9.8)	34/2,310 (1.5)	39/2,376 (1.6)	181/2,310 (7.8)	179/2,376 (7.5)	12/2,310 (0.5)	14/2,376 (0.6)	4/2,310 (0.2)	2/2,376 (0.1)	9/2,310 (0.4)	14/2,376 (0.6)	8/2,310 (0.3)	14/2,376 (0.6)
	No	105/919 (11.4)	98/858 (11.4)	8/919 (0.9)	12/858 (1.4)	93/919 (10.1)	90/858 (10.5)	15/919 (1.6)	8/858 (0.9)	5/919 (0.5)	4/858 (0.5)	2/919 (0.2)	3/858 (0.3)	2/919 (0.2)	3/858 (0.3)
MRA	Yes	128/1,288 (9.9)	139/1,259 (11.0)	16/1,288 (1.2)	18/1,259 (1.4)	113/1,288 (8.8)	121/1,259 (9.6)	6/1,288 (0.5)	8/1,259 (0.6)	1/1,288 (0.1)	1/1,259 (0.1)	4/1,288 (0.3)	4/1,259 (0.3)	3/1,288 (0.2)	4/1,259 (0.3)
	No	199/1,941 (10.3)	192/1,975 (9.7)	26/1,941 (1.3)	33/1,975 (1.7)	161/1,941 (8.3)	148/1,975 (7.5)	21/1,941 (1.1)	14/1,975 (0.7)	8/1,941 (0.4)	5/1,975 (0.3)	7/1,941 (0.4)	13/1,975 (0.7)	7/1,941 (0.4)	13/1,975 (0.7)
Loop diuretics	Yes	130/1,065 (12.2)	142/1,123 (12.6)	15/1,065 (1.4)	17/1,123 (1.5)	116/1,065 (10.9)	125/1,123 (11.1)	17/1,065 (1.6)	10/1,123 (0.9)	3/1,065 (0.3)	3/1,123 (0.3)	3/1,065 (0.3)	7/1,123 (0.6)	2/1,065 (0.2)	7/1,123 (0.6)
	No	197/2,164 (9.1)	189/2,111 (9.0)	27/2,164 (1.2)	34/2,111 (1.6)	158/2,164 (7.3)	144/2,111 (6.8)	10/2,164 (0.5)	12/2,111 (0.6)	6/2,164 (0.3)	3/2,111 (0.1)	8/2,164 (0.4)	10/2,111 (0.5)	8/2,164 (0.4)	10/2,111 (0.5)

AEs among patients in EMPACT-MI who received at least one dose of empagliflozin or placebo according to key subgroups. Shown are AEs analyzed up to 7 days after discontinuation of the trial regimen. AEs reported in the trial included serious AEs, AEs that led to discontinuation of the trial regimen for at least 7 days and AEs of special interest, defined as ketoacidosis, and AEs leading to lower-limb amputation, hepatic injury and contrast-induced kidney injury. ^a^Events identified using a MedDRA v.26.1 query. ^b^Events identified using a Boehringer Ingelheim-customized MedDRA v.26.1 query.

The rates of individual AEs within 30 days after the first study drug intake between patients randomized to empagliflozin or placebo were similar, and overall low, including serious AEs (8.3% versus 8.5%), AEs leading to permanent treatment discontinuation (1.6% versus 1.3%), acute renal failure (0.7% versus 0.8%), contrast-induced AKI (0.2% versus 0.3%), volume depletion (0.5% versus 0.3%) and hypotension (0.5% versus 0.3%) (Table 3). Results were consistent among the aforementioned subgroups (Table 3) and across the subgroups of no use, prior use and new introduction of ACEi/ARB/ARNi, MRA and loop diuretics during the index hospitalization (Supplementary Tables 5–7).

Of note, higher rates of serious AEs and acute renal failure within 30 days after the first study drug intake were observed in certain vulnerable subgroups, such as those with lower eGFR (<60 ml min^−1^ 1.73 m^−^^2^) at baseline and lower baseline SBP (<110 mmHg), in both empagliflozin and placebo arms, compared to those in the lower-risk groups (Table 3). Irrespectively, the rates of these events were numerically lower in the empagliflozin group compared to placebo.

Overall, the incidence rates of any AE over the entire study period were similar in both the empagliflozin and placebo groups (27.6% versus 27.3%) and for serious AEs (23.7% versus 24.7%). Additionally, the rates of any AEs and serious AEs showed an increasing frequency trend with lower baseline eGFR, although consistently in both treatment arms (Supplementary Table 8). Of note, the rates of acute renal failure were generally numerically lower in the empagliflozin group.

## Discussion

In patients with acute MI and increased risk for HF, empagliflozin had kidney-protective effects by reducing the decline in eGFR. Empagliflozin decreased the rate of HF endpoints, and these findings were consistent irrespective of baseline kidney function. Initiation of empagliflozin therapy shortly after acute MI was safe, with similar AE rates in the empagliflozin and placebo groups, regardless of baseline kidney function, blood pressure or use of clinically relevant concomitant therapies.

In clinical practice, physicians may worry about starting SGLT2 inhibitors because of an acute decrease in eGFR, as patients with acute MI are a vulnerable population because of high rates of contrast exposure and AKI^6^. However, this study shows that the acute treatment effect on eGFR with empagliflozin was similar to placebo. After an initial decline in eGFR at 2 weeks, eGFR fully recovered among patients in the empagliflozin group; in the long term, it decreased among patients in the placebo group. This initial change in eGFR has previously been observed with SGLT2 inhibitors; it is hemodynamic and therefore non-harmful in the long term^19–23^. Among the subgroups, an initial eGFR decline with empagliflozin and placebo was observed in patients with an eGFR ≥60 ml min^−1^ 1.73 m^−^^2^ but not among those with an eGFR <60 ml min^−1^ 1.73 m^−^^2^. Previous studies of empagliflozin showed a subgroup of patients who had increases in eGFR after treatment initiation, although these patients did not sustain these improvements in eGFR in a longitudinal follow-up^22,23^. Kidney-protective effects with SGLT2 inhibitors have been demonstrated in other populations^19,24–27^ but not previously among patients with acute MI. In the overall population, kidney function decline is more prevalent among patients with prior cardiovascular events^28,29^; thus, the stability in eGFR observed in this study with empagliflozin further supports the use of this therapy in this population. The observed patterns in eGFR change were consistent according to baseline kidney function and the use of ACEi/ARB/ARNi therapy, MRAs or loop diuretics. Other kidney outcomes, such as AKI, had numerically lower rates with empagliflozin, although low event rates limit the interpretation of these results.

While SGLT2 inhibitors are known to reduce HF events among patients with CKD, HF or T2D and high CV risk^30–36^, EMPACT-MI was the first study to show the benefit in risk reduction for HF in patients with acute MI^2^. This study furthers these data by demonstrating that the HF benefit is consistent across several levels of baseline kidney function^1,2^. Notably, patients with CKD are at especially high risk of HF; thus, therapies to reduce this burden could have substantial impact^28^. The rates of all-cause mortality were comparable. The rates of key concomitant medical therapies at baseline. such as ACEi/ARB/ARNi therapy, MRAs or loop diuretics were similar among patients in the ≥90, ≥60 to <90 and ≥30 to <60 ml min^−1^ 1.73 m^−^^2^ eGFR subgroups overall and across the empagliflozin and placebo groups. Not surprisingly, rates of ACEi/ARB/ARNi and MRA use were lower among those with an eGFR <30 ml min^−1^ 1.73 m^−^^2^, probably because of lower renal function; rates of loop diuretic use were higher, probably from increased risk of congestion with advanced kidney disease, with comparable rates in the empagliflozin and placebo groups. As this study included patients with increased HF risk, defined by newly developed LVEF of less than 45% or signs or symptoms of congestion requiring treatment, future studies to assess the benefits in patients with acute MI not at especially high HF risk will be important, as this remains an understudied population.

Our study further supports the safety of initiating empagliflozin during or early after index hospitalization for index acute MI. Overall, AE rates were similar between the empagliflozin and placebo groups during the initial 30-day period after the first study drug intake and over the entire study period across a variety of clinically important subgroups, such as those categorized according to baseline eGFR and baseline SBP. Importantly, the observed rates of acute renal failure were consistently lower in the empagliflozin group compared to the placebo group in the vulnerable subgroups with lower baseline eGFR (<60 ml min^−1^ 1.73 m^−^^2^) and lower baseline SBP (<110 mmHg).

We observed high rates of the initiation of key therapies such as ACEi/ARB/ARNi, MRA or loop diuretics at hospitalization for index acute MI. Regardless, AEs were comparable among those with baseline use of ACEi/ARB/ARNi, MRAs or loop diuretics, and among patients initiating these therapies.

We acknowledge the limitations of these analyses. Longitudinal eGFR data were only requested from patients in a subset of countries. As the current trial follows an event-driven study design where patients are randomized at different time points and are therefore followed for a different length of time until termination of the trial, only a smaller subset of these patients had eGFR data available at the 24-month time point. The number of patients who are expected to provide an eGFR measurement, defined as those who are still alive and at the follow-up in the trial at the respective time points is provided in Fig. 1. The sample size of patients with an eGFR <30 ml min^−1^ 1.73 m^−^^2^ was small. EMPACT-MI was not powered for kidney outcomes that may have limited power for these endpoints. This study only included patients with acute MI and a newly developed LVEF of less than 45% or signs or symptoms of congestion requiring treatment. Because the urine albumin:creatinine ratio was not collected at baseline or after randomization, baseline data for this biomarker could not be reported and analyses for changes in this biomarker could not be performed. Among patients with longitudinal eGFR data, eGFR was marginally lower at baseline in the empagliflozin than in the placebo group (75.2 versus 73.2 ml min^−1^ 1.73 m^−^^2^), although the models accounted for age and baseline eGFR and evaluated changes in eGFR from baseline, probably minimizing the impact of this difference.

In conclusion, empagliflozin demonstrated beneficial kidney-protective effects, reduced HF outcomes and was safe to initiate soon after acute MI. The cardiovascular–kidney benefits and safety of empagliflozin were consistent irrespective of baseline kidney function.

## Methods

### Ethics statement

EMPACT-MI was approved by the ethics committees at each clinical site; all patients provided written informed consent. The trial was registered at ClinicalTrials.gov (NCT04509674).

### Study design

EMPACT-MI was a double-blind, multicenter, placebo-controlled clinical trial, which has been described previously^1,3,37^. Inclusion criteria included age 18 years or older, hospitalization for acute MI within 14 days of admission and either a newly developed LVEF of less than 45% or treatment for congestion during the index hospitalization (or both). Additional inclusion criteria included at least one risk factor for hospitalization for HF (for example, new LVEF of less than 35%, history of MI, eGFR of less than 60 ml min^−1^ 1.73 m^−^^2^). Patients with a history of HF, eGFR of less than 20 ml min^−1^ 1.73 m^−^^2^ or requiring dialysis were excluded. Patients were randomized according to a 1:1 ratio to empagliflozin 10 mg daily or placebo in addition to standard of care therapies, with randomization stratified according to T2D history and geographical region. Randomization occurred with an interactive response technology; assignment was based on a computer-generated random sequence. Patients and investigators were blinded to treatment allocation. Access to randomization codes was monitored and restricted. Study staff who enrolled patients were blinded to the treatment group. Full inclusion and exclusion criteria, and study procedures, have been described previously^1^.

Details of the prespecified and post-hoc subgroups are presented in Supplementary Table 9. Patients were categorized into prespecified baseline eGFR categories, with categories including eGFR ≥90 ml min^−1^ 1.73 m^−^^2^, ≥60 to <90 ml min^−1^ 1.73 m^−^^2^, ≥30 to <60 ml min^−1^ 1.73 m^−^^2^ and <30 ml min^−1^ 1.73 m^−^^2^. Longitudinal eGFR monitoring was conducted for a subset of patients only, specifically those treated in Bulgaria, Germany, Hungary and Serbia, as per requirement of local health authorities. eGFR was collected at the screening, randomization, 2-week, 6-month and end-of-study visits. For analyses requiring longitudinal eGFR data, only these patients were included, and referred to as the eGFR longitudinal subset in the following. eGFR was determined using the CKD-EPI equation^38^.

### Endpoints

Details on prespecified and post hoc endpoints are presented in Supplementary Table 9. The prespecified kidney endpoint included changes in eGFR from baseline. This was evaluated over time, including at 2 weeks and at 24 months. Additional post-hoc kidney endpoints included time-to-first endpoint of kidney disease progression, AKI, a composite of kidney disease progression or all-cause mortality, and a composite of AKI, chronic renal replacement therapy, renal transplantation or renal death. Changes in eGFR over time were evaluated until 24 months (including prespecified changes until 6 months) during the time on treatment and on the study. Changes in eGFR and the first kidney disease progression endpoint were only available for the eGFR longitudinal subset. Kidney disease progression was defined as a decline of eGFR of 40% or greater from baseline, an eGFR <15 ml min^−1^ 1.73 m^−^^2^ for patients with a baseline eGFR ≥30 ml min^−1^ 1.73 m^−^^2^ or an eGFR of <10 ml min^−1^ 1.73 m^−^^2^ for patients with a baseline eGFR <30 ml min^−1^ 1.73 m^−^^2^. Renal death was defined as a death event with primary AE leading to death by narrow standardized MedDRA query of ‘acute renal failure’. Chronic renal replacement therapy was defined as renal replacement therapy lasting for at least 90 days.

The primary prespecified endpoint of EMPACT-MI was the composite of time-to-first-hospitalization for heart failure or all-cause mortality. There was no central adjudication performed in this trial. Trained investigators determined the outcomes based on prespecified definitions. Investigators were blinded to treatment group. An additional prespecified HF endpoint included the total number of hospitalizations for HF and time to all-cause mortality. Post-hoc endpoints included a composite of the total number of AEs of HF or all-cause mortality, and separately the total number of AEs of HF. AE of HF was a broader endpoint definition that included the prespecified endpoint of HF hospitalization and investigator-reported AEs categorized as ‘cardiac failure’ (including non-fatal AEs), with ‘cardiac failure’ determined according to narrow standardized MedDRA query (www.meddra.org/)^2^.

### Safety

AEs that were to be reported in this clinical trial were defined as investigator-reported serious AEs, AEs leading to trial drug discontinuation for 7 or more days and AEs of special interest (that is, contrast-induced AKI, ketoacidosis, events leading to lower-limb amputation and hepatic injury). Multiple safety outcomes were investigated, including any AE (to be reported), AE leading to permanent treatment discontinuation, serious AE and other AEs of interest—acute renal failure (to be reported), contrast-induced AKI, volume depletion (to be reported) and hypotension (to be reported).

To assess the safety of initiating therapy, multiple distinct subgroups of interest were identified. Patients were stratified according to baseline eGFR, baseline SBP and use of kidney-function-modifying therapies, specifically ACEi/ARB/ARNi therapy, MRAs and loop diuretics. We classified patients according to the baseline use of these medications and in additional analyses according to the use of these medications into groups of ‘use at discharge and start prior to index hospitalization’, ‘use at discharge and initiation of medication during the index hospitalization’ and ‘no use of medication at discharge’.

Safety outcomes were assessed within 30 days from the first study drug dose to capture the events most affected by therapy initiation, as well as through the entire study period up to 7 days after the discontinuation of the trial regimen.

### Statistical analyses

Patients were categorized into eGFR categories and baseline characteristics were presented. Time-to-first-event endpoints (for example, all-cause mortality) were assessed with Cox proportional hazards models. Relative and absolute differences in total event endpoints (first and recurrent) between the empagliflozin and placebo groups were assessed using negative binomial regression models. To assess heterogeneity in treatment effect according to eGFR category, an interaction term for treatment and baseline eGFR category was included in the model. Models included adjustment for the baseline covariates of age, sex, geographical region, eGFR, LVEF (<35% or ≥35%), T2D status, persistent or permanent atrial fibrillation, previous MI, peripheral artery disease and smoking status, and including the logarithm of time as an adjustment for the observation time. Notably, sex was added as an additional factor to the primary model compared to prior reports^1^.

Changes in eGFR over time on treatment and during the study were assessed with mixed models to account for repeated eGFR measurements, with models adjusted for age as a linear covariate and sex, geographical region, baseline LVEF, T2D status, persistent or permanent atrial fibrillation, previous MI, peripheral artery disease, smoking status, baseline eGFR according to visit and visit according to treatment interactions as fixed effects. An unstructured covariance structure was used to model within-patient errors. To evaluate eGFR over time on treatment across subgroups of baseline use of ACEi/ARB/ARNi therapy, MRAs and loop or high-ceiling diuretics, baseline SBP and baseline eGFR, an additional factor for the subgroup according to visit and according to treatment interaction was added as a fixed effect. Changes in eGFR until last value on treatment and last value in study were assessed with the analysis of covariance models adjusted for age as a linear covariate and sex, geographical region, baseline LVEF, T2D status, persistent or permanent atrial fibrillation, previous MI, peripheral artery disease, smoking status and baseline eGFR. Safety outcomes were analyzed descriptively.

No patients had missing baseline eGFR data. Data for patients who did not have an endpoint event were censored on the last day they were known to be event-free. All analyses were conducted with SAS v.9.4 (SAS Institute, www.sas.com/en_gb/home.html). No correction for multiple testing was performed; a *P* < 0.05 was deemed statistically significant.

### Reporting summary

Further information on research design is available in the Nature Portfolio Reporting Summary linked to this article.

## Supplementary information

Reporting Summary

Supplementary Tables 1–9 Supplementary Table 1: Baseline characteristics by kidney function and treatment group. Supplementary Table 2: Baseline characteristics of longitudinal eGFR subgroup overall and by treatment group. Supplementary Table 3: Change in kidney function at last value on-treatment and last value in study. Supplementary Table 4: Change in kidney function at last value in study. Supplementary Table 5: Adverse events within 30 days after first dose of study drug by ACEi/ARB/ARNi use at index hospitalization. Supplementary Table 6: Adverse events within 30 days after first dose of study drug by MRA use at index hospitalization. Supplementary Table 7: Adverse events within 30 days after first dose of study drug by loop diuretics use at index hospitalization. Supplementary Table 8: Adverse events by baseline eGFR categories. Supplementary Table 9: Pre-specification of outcomes and statistical analyses in the EMPACT-MI trial

## Data Availability

To ensure the independent interpretation of clinical study results and enable authors to fulfill their role and obligations under the International Committee of Medical Journal Editors (ICMJE) criteria, Boehringer Ingelheim grants all external authors access to relevant clinical study data. In adherence with the Boehringer Ingelheim Policy on Transparency and Publication of Clinical Study Data, scientific and medical researchers can request access to clinical study data typically 1 year after the approval has been granted by major regulatory authorities or after termination of the development program. Researchers should use the Vivli (https://vivli.org/) link to request access to the study data and visit https://www.mystudywindow.com/msw/datasharing for further information.
