# Supplementary material for: Secondary analysis of the EMPACT-MI trial reveals cardiovascular–kidney efficacy and safety of empagliflozin after acute myocardial infarction
Source: Nat Cardiovasc Res. 2025 Jun 13;4(6):761–72. doi: 10.1038/s44161-025-00657-7 (PMC12170341; doi:10.1038/s44161-025-00657-7)
Supplement: Supplementary file 1 — Reporting Summary [file 44161_2025_657_MOESM1_ESM.pdf]

nature portfolio

Corresponding author(s): Dr. Deepak L. Bhatt

Last updated by author(s): 04/08/2025

Reporting Summary

Nature Portfolio wishes to improve the reproducibility of the work that we publish. This form provides structure for consistency and transparency in reporting. For further information on Nature Portfolio policies, see our Editorial Policies and the Editorial Policy Checklist.

Statistics

For all statistical analyses, confirm that the following items are present in the figure legend, table legend, main text, or Methods section.

n/a	Confirmed	
☐	☒	The exact sample size (n) for each experimental group/condition, given as a discrete number and unit of measurement
☒	☐	A statement on whether measurements were taken from distinct samples or whether the same sample was measured repeatedly
☐	☒	The statistical test(s) used AND whether they are one- or two-sided<br>*Only common tests should be described solely by name; describe more complex techniques in the Methods section.*
☐	☒	A description of all covariates tested
☐	☒	A description of any assumptions or corrections, such as tests of normality and adjustment for multiple comparisons
☐	☒	A full description of the statistical parameters including central tendency (e.g. means) or other basic estimates (e.g. regression coefficient) AND variation (e.g. standard deviation) or associated estimates of uncertainty (e.g. confidence intervals)
☐	☒	For null hypothesis testing, the test statistic (e.g. F, t, r) with confidence intervals, effect sizes, degrees of freedom and P value noted<br>*Give P values as exact values whenever suitable.*
☒	☐	For Bayesian analysis, information on the choice of priors and Markov chain Monte Carlo settings
☒	☐	For hierarchical and complex designs, identification of the appropriate level for tests and full reporting of outcomes
☐	☒	Estimates of effect sizes (e.g. Cohen's d, Pearson's r), indicating how they were calculated

Our web collection on statistics for biologists contains articles on many of the points above.

Software and code

Policy information about availability of computer code

Data collection	Medidata Rave EDC, last used Version was10-Nov-2023. Medidata Patient Cloud Android, 2023.2.3 as of 13-Nov-2023, Medidata Patient Cloud iOS 2023.2.5 as of13-Nov-2023, Medidata Rave eConsent 2023.1.2 as of30-May-2023.
Data analysis	All analyses were conducted with SAS version 9.4.

For manuscripts utilizing custom algorithms or software that are central to the research but not yet described in published literature, software must be made available to editors and reviewers. We strongly encourage code deposition in a community repository (e.g. GitHub). See the Nature Portfolio guidelines for submitting code & software for further information.

Data

Policy information about availability of data

All manuscripts must include a data availability statement. This statement should provide the following information, where applicable:

- Accession codes, unique identifiers, or web links for publicly available datasets
- A description of any restrictions on data availability
- For clinical datasets or third party data, please ensure that the statement adheres to our policy

To ensure independent interpretation of clinical study results and enable authors to fulfil their role and obligations under the ICMJE criteria, Boehringer Ingelheim grants all external authors access to relevant clinical study data. In adherence with the Boehringer Ingelheim Policy on Transparency and Publication of Clinical Study Data, scientific and medical researchers can request access to clinical study data, typically, one year after the approval has been granted by major Regulatory

Authorities or after termination of the development program. Researchers should use the https://vivli.org/ link to request access to study data and visit https://www.mystudywindow.com/msw/datasharing for further information.

Human research participants

Policy information about studies involving human research participants and Sex and Gender in Research.

Reporting on sex and gender	The information about sex was collected based on patient medical documentation. The trial was designed in the way to have proportion of sexes in the trial to be representative to real world population of patients with acute MI. The efficacy and safety outcomes of the trial have been analysed according to sex and the results of this sex-related analysis will be published in separate paper and presented in one of global congresses
Population characteristics	The median time from index acute MI to randomization was 5 days (interquartile range [IQR]: 3-8 days) and median duration of follow-up was 17.9 months. Median time from hospital admission for index MI to discharge was 5 days (3, 8 days). Mean age was 63.6 years (standard deviation [sd]: 10.9 years), with 1625 (24.9%) females. Among these patients, mean eGFR was 76.1 ml/min/1.73 m2 (sd: 19.9 ml/min/1.73 m2). A total of 1803 (27.6%) had eGFR ≥90 mL/min/1.73 m2, 3261 (50.0%) 60-<90 mL/min/1.73 m2, 1399 (21.5%) 30-<60 mL/min/1.73 m2, and 59 (0.9%) <30 mL/min/1.73 m2. A total of 4845 (74.3%) patients presented with ST-elevation MI (STEMI) and 1675 (25.7%) with non-ST-elevation MI (NSTEMI), with 5822 (89.3%) patients who underwent revascularization. A total of 1886 (28.9%) patients received additional contrast administration in addition to coronary angiography or revascularization procedure.
Recruitment	The patients were recruited at 451 clinical sites across 22 countries. The recruitment continued from Dec 2020 to March 2023
Ethics oversight	EMPACT MI was approved by the ethics committees at each clinical site and all patients provided written informed consent. The trial was registered at ClinicalTrials.gov (NCT04509674).

Note that full information on the approval of the study protocol must also be provided in the manuscript.

Field-specific reporting

Please select the one below that is the best fit for your research. If you are not sure, read the appropriate sections before making your selection.

☒ Life sciences ☐ Behavioural & social sciences ☐ Ecological, evolutionary & environmental sciences

For a reference copy of the document with all sections, see nature.com/documents/nr-reporting-summary-flat.pdf

Life sciences study design

All studies must disclose on these points even when the disclosure is negative.

Sample size	6522 patients. Sample size was determined by including all patients in EMPACT-MI. Sample size is sufficient as this study is a pre-specified analysis of patients enrolled in the trial
Data exclusions	Patients with a history of HF, eGFR of less than 20 mL/min/1.73 m2, or requiring dialysis were excluded. Full inclusion and exclusion criteria, as well as study procedures, have previously been published [Butler J et al. N Engl J Med. 2024;390:1455–1466]
Replication	The outcomes evaluated in the presented work have been previously evaluated in other large-scale clinical trials of empagliflozin in adjacent patient populations (EMPEROR-Reduced, EMPEROR-Preserved, EMPA-REG OUTCOME, EMPA-KIDNEY)
Randomization	Patients were randomized in a 1:1 ratio to empagliflozin 10mg daily or placebo. Patient assignment to a treatment group was determined by a computer-generated random sequence. The randomisation list was generated using a validated system, which involved a pseudo-random number generator so that the resulting treatment was both reproducible and non-predictable
Blinding	All investigators and study participants were blinded for group allocation

Reporting for specific materials, systems and methods

We require information from authors about some types of materials, experimental systems and methods used in many studies. Here, indicate whether each material, system or method listed is relevant to your study. If you are not sure if a list item applies to your research, read the appropriate section before selecting a response.

Materials & experimental systems

n/a	Involved in the study
☒	☐ Antibodies
☒	☐ Eukaryotic cell lines
☒	☐ Palaeontology and archaeology
☒	☐ Animals and other organisms
☐	☒ Clinical data
☒	☐ Dual use research of concern

Methods

n/a	Involved in the study
☒	☐ ChIP-seq
☒	☐ Flow cytometry
☒	☐ MRI-based neuroimaging

Clinical data

Policy information about clinical studies

All manuscripts should comply with the ICMJE guidelines for publication of clinical research and a completed CONSORT checklist must be included with all submissions.

Clinical trial registration	The trial was registered at ClinicalTrials.gov (NCT04509674).
Study protocol	The full trial protocol has been published along with primary trial publication: Butler J et al. N Engl J Med. 2024;390:1455–1466
Data collection	EMPACT-MI was prospective clinical trial conducted from Dec 2020 to Nov 2023 with recruitment period from Dec 2020 to March 2023 in 451 clinical sites in 22 countries: Romania, Poland, Ukraine, Bulgaria, Israel, Serbia, the Netherlands, Germany, Hungary, Russia, France, Spain, Denmark, Australia, USA, Canada, India, Japan, China, Republic of Korea, Argentina, Brazil.
Outcomes	Time to first hospitalization for heart failure (HHF) or all-cause mortality. Total number of HHF and time to all-cause mortality. Total number of adverse events (AE) of HF or all-cause mortality. Total number of AEs of HF Changes in eGFR from baseline, time-to-first endpoints of kidney disease progression, AKI, a composite of kidney disease progression or all-cause mortality, and a composite of AKI, chronic renal replacement therapy, renal transplantation, or renal death. Relative and absolute differences in total event endpoints (first and recurrent) between the empagliflozin and placebo group were assessed with negative binomial regression models. Changes in eGFR over time on-treatment and during the study were assessed with mixed models.
